# Activation of innate and adaptive immunity as an effective combined strategy for cancer immunotherapy

**DOI:** 10.1186/2051-1426-3-S2-P370

**Published:** 2015-11-04

**Authors:** Alexander Rakhmilevich, Mildred Felder, Lauren Lever, Tyler Van De Voort, Alan J Korman, Stephen D Gillies, Paul M Sondel

**Affiliations:** 1 UW-Madison, Madison, WI, USA; 2 Bristol-Myers Squibb Company, Redwood City, CA, USA; 3 Provenance Biopharmaceuticals, Carlisle, MA, USA; 4 Department of Human Oncology, Department of Pediatrics, University of Wisconsin-Madison, Madison, WI, USA

## 

Immunotherapeutic approaches can demonstrate some antitumor benefit, but their efficacy is limited when they are used as a single modality. We asked if a combinatory approach activating both innate and adaptive immunity would improve cancer immunotherapy. We have previously shown that an agonistic anti-CD40 monoclonal antibody (anti-CD40) in combination with a toll-like receptor 9 agonist, CpG, can activate macrophages in mice, leading to tumor cell killing. Separately, we have shown that a direct intratumoral injection of an immunocytokine (IC) consisting of anti-GD2 antibody linked to interleukin-2 can activate NK and T cells, resulting in antitumor effects. We hypothesize that activation of macrophages (with anti-CD40/CpG) and NK cells (with IC) will increase tumor destruction and presentation of tumor antigens, leading to T cell activation, which in turn could be further augmented by anti-CTLA-4 antibody, resulting in tumor eradication and prevention of tumor recurrence. Using the mouse GD2^+^ B78 melanoma model, we show that anti-CD40/CpG and IC/anti-CTLA-4 synergistically induced regression of established subcutaneous tumors, resulting in the cure of 50% of mice and development of immunological memory against B78 as well as wild type B16 tumors. While the antitumor effect of anti-CD40/CpG was T cell independent, the antitumor effect of IC/anti-CTLA-4 required T cells. Anti-CD40/CpG treatment led to upregulation of T cell activation markers in draining lymph nodes. Finally, the combined treatment with anti-CD40/CpG and IC/anti-CTLA-4 was effective against B16 lung metastases. We suggest that a combination of anti-CD40/CpG and IC/anti-CTLA-4 should be tested as a clinically relevant novel treatment strategy.

